# Supplementary material for: Hepatitis B and C viral coinfection and associated factors among HIV-positive patients attending ART clinics of Afar regional state, northeast Ethiopia
Source: PLoS One. 2024 May 16;19(5):e0302453. doi: 10.1371/journal.pone.0302453 (PMC11098400; doi:10.1371/journal.pone.0302453)
Supplement: S1 File — (DOCX) [file pone.0302453.s001.docx]

# ANNEXES

## Annex I: Information Sheet

**Name of researching organization(s):** Mekelle University College of Health Science

**Title of the project**: Seroprevalence of HBV and HCV co-infection and associated risk factors among HIV positive patients attending ART clinics at selected health facilities, Afar, North Eastern Ethiopia.

**Research objective:** The purpose of this study is to assess seroprevalence of HBV and HCV co-infection and associated risk factors among HIV positive patients attending ART clinics at selected health facilities, Afar, North Eastern Ethiopia from February 1 to April 30, 2019**.**

**Significance of the study:** Because of your participation, prevalence and risk factors of hepatitis B and C co infections among HIV positive patients attending ART clinics of health facilities, Afar, north east Ethiopia will be determined. Therefore your participation is essential to implement preventive interventions and strategic policy directions including systematic screening of all newly diagnosed HIV cases for co infection to improve management strategies for HBV and HCV infection and antiretroviral therapy (ART) implementation.

**Budget:** covered by the principal investigator.

**Investigator:** Yemane Mengsteab (MSC student on Medical microbiology and immunology) and assigned advisors from Mekele Unversity College of Health Science (MUCHS) unit of Medical microbiology and immunology

**Study procedure:** To achieve the planned objective, a standardized questionnaire will be used to collect the socio-demographic and risk factors for hepatitis B and C co infections among HIV positive patients attending ART clinics. Then after, venous blood sample will be collected from each participating individuals and laboratory examinations will be performed for detecting HBsAg and anti HCV.

**Risks:** There will not be risk associated during sample collection and after.

**Participant’s role:** If you agree to participate, you will be asked to give personal identification information (age, sex, address, etc) and concerning risk factors for hepatitis B and hepatitis C co infection among HIV positive patients attending ART clinics. In addition, your will give a venous blood sample.

**Participant’s right:** Since participation is on volunteer bases, participant has the right to participate in study and can withdraw from participation under any condition. You have full right to refuse to answer questionnaire and/or to give the requested samples. Nothing will happen to you because of your refusal to participate.

**Benefit:** You will not be charged for all the laboratory diagnosis of HBV and HCV and you will have full right to know your sero status of these viruses.

**Incentives:** There are no special incentives because of participation. Participation is your free choice.

**Confidentiality:** Your answers to interview will be registered in questionnaire format. Your name will not be mentioned in any report, instead code will be used. All of your answers and test results will keep confidential and will not be given to other institution and/ or person except for the principal investigator of this study. Your information will be used only for above mentioned purpose**.**

**Agreement:** You will be asked to sign for signature of agreement. This is to make sure that your agreement to participate in the mentioned study is on volunteer and informed bases. Otherwise there is no other reason for signing. The study is approved by Mekelle University, College of health sciences ethical review committee. Getting signatures of agreement from participant is one of the criteria of the committee for the indication of no one can participate in the study without participant consent and agreement. Participants will make agreements on their volunteer bases. You have full right to get full information about study procedures and other related issues with languages of your choice.

**Whom to contact:** If you have any question or doubt you can contact:-

Yemane Mengsteab (Mekelle University, College of Health Science Department of Immunology and Microbiology)

Tel: +251922919918

E-mail: mengsteaby40@ gmail.com

## Annex II: Informed Consent

**Name of the research institute**: Mekelle University College of Health Sciences

**Principal investigator**: Yemane Mengsteab

**Research topic**: Seroprevalence of HBV and HCV co-infection and associated risk factors among HIV positive patients attending ART clinics at selected health facilities, Afar, North Eastern Ethiopia.

As the investigator has explained me/ as I have understood from the information sheet, my participation is worthy and I decided to participate in this study and to give all the necessary data and sample. I would like to confirm you based on my signature.

ID number of the participant ____________________signature ________Date ______________

Name of investigator _____________________Signature ____________Date______________

Thank you again!

## Annex III: Questionnaire

Questionnaire for Seroprevalence and associated risk factors of hepatitis B and C virus co-infections among HIV positive patients attending ART clinics at selected health facilities, Afar, North Eastern Ethiopia..

Patient code No (given by principal investigator) __________________

1. **Identification**
   - - 1. Patient MR No _____________
       2. Age in years ______________
       3. Sex

Male Female

-

1. **Socio-demographic and ART status and CD 4 data**
2. Residence

Rural Urban

1. Marital status

Single Married

Divorced Widowed

1. Educational level

Illiterate Elementary

Secondary Certificate and above

1. Occupation

Farmer Merchant

Civil servant Driver

Housewives Commercial sex worker

No work others (specify) -------------------

1. Most recent CD_4_ count

<200 200–350

351–499 ≧500

1. ART status

On ART Pre-ART

1. **Questions related to associated risk factors**

Have you have or ever practiced the following

- - - 1. Multiple sexual partner No Yes
      2. Blood transfusion recipient No Yes
      3. Abortion history No Yes
      4. Hospital admission history No Yes
      5. Surgical procedure No Yes

6. Home delivery No Yes

1. Dental procedure No Yes
2. Female genital mutilation No Yes
3. Tattooing No Yes
4. Piercing No Yes
5. Scarification No Yes

1. Household contact with Jaundice No Yes
