# Supplementary material for: Hepatitis B and C viral coinfection and associated factors among HIV-positive patients attending ART clinics of Afar regional state, northeast Ethiopia
Source: PLoS One. 2024 May 16;19(5):e0302453. doi: 10.1371/journal.pone.0302453 (PMC11098400; doi:10.1371/journal.pone.0302453)
Supplement: S2 File — (PDF) [file pone.0302453.s002.pdf]

Mekelle University
College of Health Sciences
Health Research Ethics Review Committee (HRERC)

To: Yemane Mengsteab
Principal Investigator
Mekelle

Date: February 8/2019
RE: Notification of Expedited Approval
ERC 1212/2019

Protocol: *Seroprevalence of HBV and HCV co-infection and associated risk factors among HIV positive patients attending ART clinics at selected Health facilities in Afar region, Northeastern Ethiopia.*

Dear PI

This is your notification that your above referenced study has received **EXPEDITED APPROVAL** on **February 8/2019.** This ethics review approval will expire on 8/2/2020.

The research study cited above has been reviewed and it has been determined that it meets the criteria for expedited review. The HRERC will be apprised of this decision at its monthly meeting.

The PI should comply with national and international scientific and ethical guidelines. Any reportable events (serious adverse events, breaches of confidentiality, protocol deviation or protocol violations) or issues resulting from this study should be reported immediately to the HRERC. Any amendments (changes to any portion of this research protocol including but not limited to protocol or informed consent changes) must have HRERC approval before being implemented.

All correspondences and inquires concerning this research protocol must include the ERC number, the name of the PI and the protocol title.

Sincerely,

Adissu Alemayehu (PhD)
Head, Research & Community Services

CC:

- BMI
- CARD

CHS

College of Health Science
Mekelle University
Institutional Ethics Review Committee

Commencing health research without approval is unethical!
