# Supplementary material for: Hepatitis B and C viral coinfection and associated factors among HIV-positive patients attending ART clinics of Afar regional state, northeast Ethiopia
Source: PLoS One. 2024 May 16;19(5):e0302453. doi: 10.1371/journal.pone.0302453 (PMC11098400; doi:10.1371/journal.pone.0302453)
Supplement: Attachment — Submitted filename: Point by point response for reviewers.docx [file pone.0302453.s003.docx]

**Point by point response for reviewers**

**Response for reviewer 1**

**Comment 1:** The paper needs to be revised for English and grammar editing (e.g., line 105-108, 184, 208)

**Response 1:** We have tried to edit grammar errors by deeply revising the manuscript and by using online editor- grammar checker.

**Comment 2:** Line 94 samara should be written in capital letter (southwest of Samara)

**Response 2:** We have written in capital letter accordingly

**Comment 3:** Line 96 and 98 clients should be changed to participants or patients.

**Response 3:** We have replaced “clients” by “patients”

**Comment 4:** Line 106: It is not clear what is meant by Source population.

**Response 4:** We have tried to make clear and easily understandable

**Comment 5:** Line 105-108-The paragraph need to be rephrased.

**Response 5:** we have rephrased the paragraph

**Comment 6:** It is not clear how long the participant was on ART and whether their medications included treatment that can treat HBV.

**Response 6:** Dear reviewer, we didn’t assess how long the participants were on ART, but we have tried to clarify the medications included that can also treat HBV.

**Comment 7:** Line 127: It is not clear why vaccinated individuals were excluded from the study since they did not check for other serological markers. Kindly clarify in the manuscript whether participants’ vaccination history was self-reported only or confirmed using clinical records. The limitations of relying on self-reported clinical history / patient recall should be adequately addressed in the manuscript where appropriate.

**Response 7:** We have modified the exclusion criteria

**Comment 8:** The authors allude to the use of a questionnaire in collecting relevant participant data. For validation purposes, kindly indicate of this questionnaire was piloted. Was a previously published questionnaire used during this study (in which case it should be appropriately cited and referenced) or if a study specific one was developed, can a template be provided as part of supplementary material.

**Response 8:** Modified and corrected

**Comment 9:** The statistical analysis is not in deep described and is not clear which statistical analysis was performed for descriptive analysis.

**Response 9:** We have tried to describe briefly the descriptive analysis (

**Comment 10:** Line 198-199: It is not clear whether the patient who tested negative by ELISA was HCV or HBV positive by rapid. The author should include the rapid results first.

**Response 10**: We have included the rapid test results and the discordant test result is clarified

**Comment 11:** Did the authors investigate whether those who have multi-sexual were commercial sex workers or not?

**Response 11:** Dear reviewer, we agreed with your idea. But we only investigate multi-sexual practice.

**Comment 12:** Table 1: p value of 0.206 in the variable scarification variables is more than 0.2; Second. & above should read secondary in the educational level variable; Indicate how many were commercial sex worker since it is a high risk for infection under the occupation variable.

**Response 12:** Accepted and Modified

**Comment 13:** Write all the abbreviations in full

**Response 13:** We have tried to write all the abbreviations in full

**Comment 14:** Line 272: It is not clear if Egypt screened blood transfusion for HBV/HCV/HIV. If it is done, when was it introduced? Did the participant indicate when they were transfused? If blood is screened for viruses there might be the reason why HCV is low.

**Response 14:** Dear reviewer, we agreed with your idea. But we only investigate the history of Blood transfusion

**Comment 15:** Line 276-282: There was no statistical significance with age and educational level.

**Response 15:** Comment accepted and modified

**Comment 16:** The discussion section should be reorganized. Discussion section should relate to the literature review and research questions and making an argument in support of the overall conclusion.

**Response 16:** We have tried to reorganize and modify it

**Response for reviewer 2**

**Comment 1:** It is recommended that the introduction should include more on the testing practice for viral hepatitis B and C in the country, some information on the vaccine policy and coverage for hepatitis B and treatment accessibility for hepatitis B and C, mono-infected and co-infected patients in the country. The added information will lead to an understanding to the findings and discussion in relation to age groups, gender and risks.

**Response 1:** we have tried to include some information on the testing, vaccine and treatment accessibility in the country

**Comment 2:** Page 3, line 44 and 46, change “peoples” to people

**Response 2:** comment accepted and corrected

**Comment 3:** Page 6, line 94, use capital “S” for Samara

**Response 3**: comment accepted and corrected

**Comment 4:** Page 7, line 112, is the estimated 6.1% prevalence for HIV? HBV? HCV? Or co-infections?

**Response 4: comment accepted and m**odified

**Comment 5:** Page 9, line 137, Is “multi-sexual practice” homosexual and heterosexual practice with multiple partners? Please may you define or provide reference to this?

**Comment 6:** Page 10, line 148, add “a” to test tube

**Response 6:** comment accepted and corrected

**Comment 7:** Page 17, line 243, “O” in “our” should be in small letter “o”.

**Response 7**: comment accepted and corrected

**Comment 8:** Page 18, line 252, our results ‘were” however….

**Response 8:** comment accepted and corrected.
